# Диагностика и тактика ведения пациента с центральным несахарным диабетом на примере клинического случая

**DOI:** 10.14341/probl13103

**Published:** 2022-07-20

**Authors:** Н. Н. Катамадзе, Е. А. Пигарова, Л. К. Дзеранова

**Affiliations:** Национальный медицинский исследовательский центр эндокринологии; Национальный медицинский исследовательский центр эндокринологии; Национальный медицинский исследовательский центр эндокринологии

**Keywords:** несахарный диабет, десмопрессин, проба с гипертоническим раствором, тест с водной депривацией

## Abstract

Диагностика и дифференциальная диагностика пациентов с несахарным диабетом нередко представляют собой сложную задачу для врачей различных специальностей. Данный клинический случай посвящен описанию пациента с длительным анамнезом идиопатического несахарного диабета центрального генеза, у которого резко снизилась потребность в десмопрессине за последний год наблюдения. Проведение проб с осмотической стимуляцией (тест с водной депривацией, инфузионная проба с гипертоническим раствором) позволило ответить на вопрос о сохранении заболевания, а также определить дальнейший план ведения с учетом физиологических особенностей.

## Abstract (en)

Diagnosis and differential diagnosis of patients with diabetes insipidus is often a difficult task for the endocrinologist. This case report focuses on a patient with a long history of central idiopathic diabetes insipidus who had a substantial decrease in desmopressin requirements during the last year of follow-up. Conducting tests with osmotic stimulation (test with water deprivation, infusion test with hypertonic solution) made it possible to answer the question of the persistence of the disease, as well as to determine a further management plan, taking into account the physiological characteristics of our patient.

## ВВЕДЕНИЕ

Центральный несахарный диабет (ЦНД) — заболевание, характеризующееся неспособностью почек реабсорбировать воду и концентрировать мочу, имеющее в своей основе дефект синтеза или секреции вазопрессина и проявляющееся выраженной жаждой и экскрецией большого количества разведенной мочи [1].

Сложная регуляция водно-электролитного обмена затрагивает различные уровни эндокринного управления. Для правильной дифференциальной диагностики требуется проведение осмотических функциональных тестов.

Мы представляем клинический случай пациента с ЦНД со снижением потребности в терапии синтетическим аналогом эндогенного антидиуретического гормона (АДГ), десмопрессином, через многие годы после манифестации заболевания илечения постоянными дозами препарата.

## ОПИСАНИЕ КЛИНИЧЕСКОГО СЛУЧАЯ

Пациент Л., 55 лет поступил в отделение нейроэндокринологии ФГБУ «НМИЦ эндокринологии» Минздрава России с жалобами на общую слабость, потливость и апатию. Считает себя больным с 1983 г. (возраст — 39 лет), когда появились жажда, полидипсия до 8 л в сутки, полиурия, снижение массы тела на 7 кг, общая слабость. При обращении к эндокринологу на основании лабораторно подтвержденной гипотонической полиурии и отсутствия данных за сахарный диабет диагностирован несахарный диабет и инициирована терапия десмопрессином в виде назальных капель (Адиуретин) по 2 капли интраназально 2 раза в сутки. Через много лет пациент был переведен на сублингвальную форму десмопрессина в эквивалентной поклинической активности суточной дозировке 120 мкг. За последние годы отмечает значимое изменение самочувствия, связанное с уменьшением потребности в препарате, это послужило основой для госпитализации с целью подтверждения ремиссии несахарного диабета.

Объективный статус: Сознание ясное. Положение активное. Масса тела 92,0 кг. Рост 172 см. Индекс массы тела 31,1 кг/м2 (ожирение I). Температура тела 36,6°С. Кожные покровы чистые, без особенностей. Склеры обычной окраски. Видимые слизистые оболочки розовые. Зев чистый. Щитовидная железа мягко-эластической консистенции, в размерах не увеличена, узловые образования не пальпируются. Перкуссия и аускультация сердца и легких без особенностей. Артериальное давление 110/70 мм рт. ст., пульс 73 в минуту. Живот при пальпации мягкий, безболезненный. St. localis: клинических признаков дегидратации не выявлено.

В базальных условиях на фоне свободного питьевого режима в биохимическом анализе крови — концентрации основных электролитных параметров в пределах референсных значений при сниженных показателях плотности и осмоляльности мочи (табл. 1 и 2). Суточный диурез на фоне приема 15 мкг десмопрессина под язык по потребности (в среднем 1 раз в сутки) составил 2,5 л.

**Table:** Таблица 1. Результаты биохимического анализа и осмоляльности крови пациентаTable 1. Results of biochemical analysis and osmolality of the patient's blood

Параметр	Показатель	Единицы измерения	Референсный интервал
Натрий	139,9	ммоль/л	136–145
Хлориды	106,1	ммоль/л	98–107
Калий	4,5	ммоль/л	3,5–5,1
Белок общий	68,6	г/л	64–83
Мочевина	4,36	ммоль/л	3,2–7,4
Креатинин	100,6	мкмоль/л	63–110
Глюкоза	5,51	ммоль/л	3,1–6,1
Фосфор	1,2	ммоль/л	0,74–1,52
Мочевая кислота	370,28	мкмоль/л	202–416
Кальций общий	2,4	ммоль/л	2,15–2,55
Альбумин	45,2	г/л	35–50

**Table:** Таблица 2. Результаты общего анализа утренней порции мочиTable 2. Results of the general analysis of the morning portion of urine

Параметр	Показатель	Единицы измерения	Референсный интервал
Относительная плотность мочи	1,005	г/мл	1,018–1,03
Осмоляльность мочи	289	мОсм/кг	600–1200
Билирубин	neg	мкмоль/л	0–8,5
Уробилиноген	norm	мкмоль/л	0–34
pH	5	-	5–6
Эритроциты	neg	в мкл	0–10
Кетоны	neg	ммоль/л	0–0,5
Нитриты	Не обнаружены	-	Не обнаружены
Лейкоциты	neg	в 1 мкл	0–25

Для подтверждения/исключения персистенции ЦНД больному проведены две пробы: проба с депривацией жидкости (сухоедением), которая является золотым стандартом для дифференциальной диагностики полиурического синдрома, и дополнительно инфузионная проба с гипертоническим (3%) раствором, целью которой также является отделение здоровых пациентов от имеющих несахарный диабет. В ходе теста с водной депривацией и инфузионной пробы с гипертоническим раствором достигнуто «плато» нарастания осмоляльности мочи до 228 и 337 мОсм/кг соответственно, в то время как лабораторные параметры осмоляльности и Na крови соответствовали состоянию обезвоживания организма с развитием гиперосмоляльности и гипернатриемии (табл. 3, 4). Учитывая выявленные изменения лабораторных параметров, диагноз несахарного диабета был подтвержден.

**Table:** Таблица 3. Результаты теста с водной депривациейTable 3. Results of the water deprivation test

Время	Вес, кг	Объем мочи, мл	АД (пульс)	Самочувствие	Натрий сыворотки крови, ммоль/л	Осмоляльность плазмы, мОсм/кг	Осмоляльностьмочи, мОсм/кг
08:30	89,9	-	100/70 (68)	Удовлетворительное	142,1	294	137
09:30	89,8	220	110/80 (80)	Незначительная жажда и сухость во рту	-	-	141
10:30	89,75	300	110/70 (70)	Незначительная жажда и сухость во рту	-	-	160
11:30	89,2	370	110/70 (70)	Незначительная жажда и сухость во рту	144,3	295	163
12:30	88,65	340	100/75 (75)	Незначительная жажда и сухость во рту	-	-	176
13:30	88,6	150	100/75 (75)	Незначительная жажда и сухость во рту	-	-	191
14:30	88,25	310	100/70 (70)	Сухость во рту, слабость	146,2	300	228

**Table:** Таблица 4. Результаты пробы с гипертоническим растворомTable 4. Results of the test with hypertonic saline

Время	АД (пульс)	Самочувствие	Натрий сыворотки крови, ммоль/л	Осмоляльность плазмы, мОсм/кг	Осмоляльность мочи, мОсм/кг
09:30	120/85 (64)	Удовлетворительное	140,1	292	212
10:00	120/80 (65)	Удовлетворительное	142,2	296	213
10:30	115/80 (60)	Удовлетворительное	143,2	297	247
11:00	115/80 (65)	Удовлетворительное	145,4	300	337
11:30	125/80 (63)	Удовлетворительное	147,6	307	-

С целью подтверждения центрального генеза несахарного диабета пациенту выполнен тест с десмопрессином (табл. 5). После приема 0,1 мг десмопрессина под язык до полного рассасывания было достигнуто значимое увеличение осмоляльности мочи на 89 и 178%, на 2 и 4 ч соответственно, что свидетельствует о наличии центральной формы заболевания.

**Table:** Таблица 5. Протокол теста с 0,1 мг десмопрессинаTable 5. Test protocol with 0.1 mg desmopressin

Время	Вес, кг	Объем мочи (мл)	АД (пульс)	Самочувствие	Осмоляльность мочи, мОсм/кг
17:00	88,3	-	120/80 (63)	Удовлетворительное	431
19:00	88,3	-	115/80 (60)	Удовлетворительное	634

С целью визуализации хиазмально-селлярной области пациенту выполнена магнитно-резонансная томография (МРТ), которая не выявила опухолевых или воспалительных образований и аномалий развития головного мозга. Гипофиз имеет нормальные размеры: вертикальный — 5 мм, поперечный — 13 мм, переднезадний — 9,1 мм. Структура аденогипофиза однородная, воронка расположена по средней линии (рис. 1, 2). Отмечено отсутствие типичного сигнала от задней доли гипофиза,а проведение контрастного усиления не выявило каких-либо дополнительных изменений гипоталамо-гипофизарной области, в связи с чем диагноз идиопатического ЦНД у пациента был подтвержден.

**Figure:**
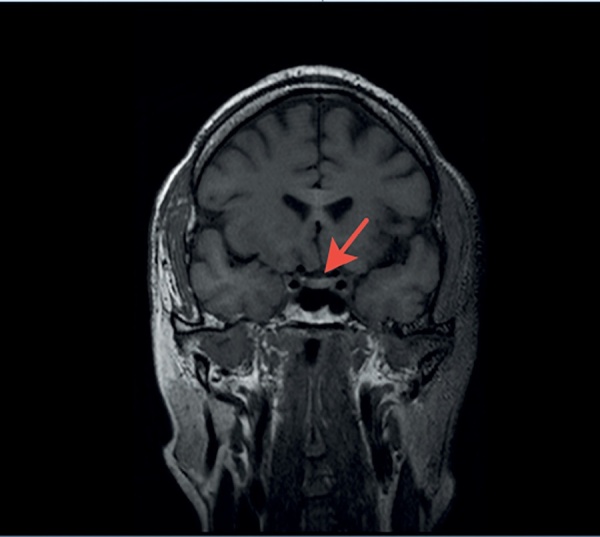
Рисунок 1. МР-картина хиазмально-селлярной области пациента. Фронтальный срез.Figure 1. MRI of the patient's chiasmal-sellar area. Front cut.

**Figure:**
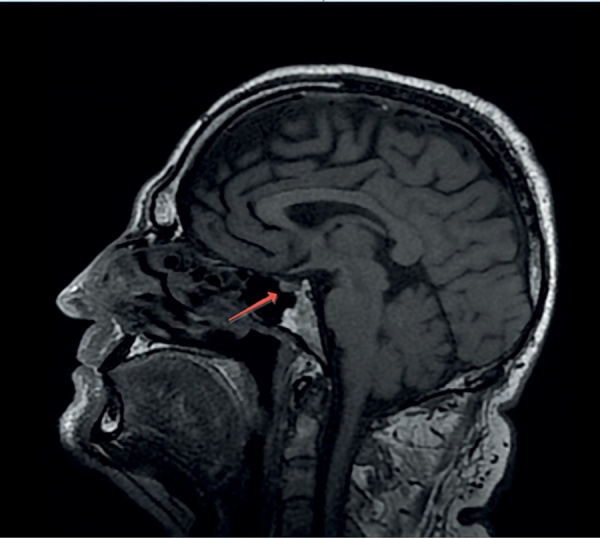
Рисунок 2. МР-картина хиазмально-селлярной области пациента. Сагиттальный срез.Figure 2. MRI of the patient's chiasmal-sellar area. Sagittal section.

## ОБСУЖДЕНИЕ

В клинической практике эндокринологов и врачей многих других специальностей возникает вопрос о дифференциальной диагностике синдрома полидипсии-полиурии. Только при последовательном соблюдении алгоритма диагностики с проведением функциональных проб возможно исключение диагноза первичной полидипсии, проявления которой часто можно спутать с симптомами несахарного диабета. Важность исключения данного диагноза обусловлена тем, что назначение любого лечения несахарного диабета, будь то десмопрессин или препараты тиазидных диуретиков и нестероидных противовоспалительных средств при первичной полидипсии, может повлечь за собой развитие водной интоксикации [1][2].

У представляемого нами пациента диагноз несахарного диабета был установлен на основании жалоб, свидетельствующих о манифестации заболевания, однако функциональный тест с осмотической стимуляцией не проводился. Учитывая отсутствие черепно-мозговых травм, оперативных вмешательств, образований гипоталамо-гипофизарной области, а также воспалительных заболеваний головного мозга при диагностике заболевания (и в последующие годы жизни) был поставлен диагноз идиопатического несахарного диабета.

Данный клинический случай примечателен тем, что через многие годы после манифестации заболевания потребность в препарате значимо снизилась до 15 мкг в сутки. Обращали на себя внимание отсутствие симптомов несахарного диабета при приеме столь низкой дозы препарата, но усиление полиурии при попытке полной отмены десмопрессина.

Наше внимание также привлекло то, что при манифестации заболевания пациенту был назначен Адиуретин (интраназальные капли десмопрессина), который до появления таблетированной формы являлся наиболее применяемым препаратом десмопрессина, однако его длительное использование сопровождалось раздражением слизистой оболочки носа. Некоторые сложности, связанные с точностью дозирования препарата, требовавшие использования назальных катетеров, затрудняли достижение компенсации заболевания [3][4].

На данный момент существует 3 наиболее распространенные формы десмопрессина. В 1987 г. была создана таблетированная форма десмопрессина, который существует в двух формах: для приема внутрь и для сублингвального применения. При приеме внутрь десмопрессин характеризуется низкой биодоступностью (от 1 до 5%), а прием с пищей снижает биодоступность еще на 40%, поэтому он обязательно должен приниматься натощак с выдерживанием интервала до приема пищи 30–40 мин или через 2 ч после еды, что не всегда удобно для соблюдения пациентами. Начальная доза — 0,1 мг 2–3 раза в сутки, далее доза подбирается в зависимости от потребностей пациента в препарате и в среднем составляет 0,1–0,2 мг 2–3 раза в сутки [5][6]. Применение сублингвальной формы препарата осуществляется путем рассасывания под языком, препарат не требуется запивать водой, для оптимизации всасывания необходимо выдержать 15-минутный интервал перед приемом пищи/едой [1]. Начальная доза составляет 60 мкг в 2–3 приема, средняя доза может составить от 60 до 960 мкг/сут [6]. Сравнительно недавно появилась форма интраназального введения десмопрессина в виде дозированного спрея. Начальная доза составляет 10 мкг (1 доза) 1–2 раза в сутки, в среднем пациентам требуется 10–40 мкг/сут. У некоторых пациентов с высокой чувствительностью к десмопрессину возможно применение интраназальных спреев всего лишь 1 раз в сутки, что может существенно влиять на комплаентность пациентов. Кроме того, возможность применения спрея в больших дозировках делает данную форму введения препарата наиболее удобной для пациентов с тяжелыми формами ЦНД [7].

Патогенез идиопатического несахарного диабета неизвестен, предполагается ведущая роль аутоиммунной агрессии к АДГ-секретирующим клеткам крупноклеточных нейронов паравентрикулярного или супраоптического ядер гипоталамуса, которая показана в научных поисковых исследованиях, но до сих пор не разработаны и не внедрены в практику диагностические наборы для подтверждения данного патологического процесса, что приводит к необходимости более частого проведения МРТ для исключения опухолевых или инфильтративных процессов, а аутоиммунный ЦНД остается при этом диагнозом исключения [8][9].

Важным параметром МРТ, высокоспецифичным для ЦНД, является снижение характерного свечения от нейрогипофиза на Т1-взвешенных изображениях. Гиперинтенсивность задней доли, наблюдаемая в норме, происходит от наличия секреторных гранул, богатых фосфолипидами, в которых содержится АДГ. При нарушении синтеза и секреции АДГ, а также в ситуациях его повышенных трат (например, декомпенсированный сахарный диабет) такой сигнал пропадает и обе доли имеют одинаковую интенсивность. Таким образом, отсутствие характерного сигнала от нейрогипофиза по данным МРТ головного мозга у представляемого нами пациента дополнительно свидетельствовало о наличии ЦНД.

Данные больших когортных исследований показывают, что в отличие от послеоперационной/посттравматической формы ЦНД, где ремиссия составляет 60–75% [7], клиническое течение заболевания в других случаях, как правило, постоянное. В литературе описаны единичные случаи пациентов с ремиссией ЦНД после многолетнего периода персистенции заболевания [10][11]. Безусловно, дальнейшие исследования патофизиологии гипоталамо-нейрогипофизарной системы необходимы для изучения патогенеза реверсивного идиопатического несахарного диабета.

Несмотря на то что представляемому пациенту исходно не проводилось функциональное тестирование, эффективность десмопрессина как исходно, так и на протяжении длительного последующего времени лечения без проявлений водной интоксикации и выявления объемных/инфильтративных изменений гипоталамо-гипофизарный области по данным МРТ безапелляционно свидетельствует о правильности постановки диагноза идиопатической формы ЦНД.

Потребность в десмопрессине у пациентов с ЦНД не зависит от параметров водного обмена, таких как объем выделенной/выпитой жидкости, уровень натрия/осмоляльность крови, а также демографических показателей или индекса массы тела. Согласно литературным данным, величина антидиуретического эффекта ограничена собственной концентрационной способностью почек человека, которая у пациентов с несахарным диабетом снижена вследствие предшествующего дефицита АДГ. Однако величина осмотического градиента почек существенно варьирует от пациента к пациенту. Любой гормон нуждается в рецепторах для обеспечения физиологической функции. Таким образом, активность гормона зависит не только от его концентрации, но и от его сродства к рецепторам-мишеням. Кроме того, количество доступных рецепторов может различаться между пациентами и подвергаться изменениям у человека с течением времени. Причиной индивидуальной чувствительности к терапии могут быть и генетические различия в базальном уровне АДГ плазмы. Хорошо известно, что с возрастом снижается высвобождение эндогенного АДГ. Таким образом, возникает вопрос: «Cледует ли за возрастным снижением высвобождения вазопрессиновых рецепторов 2 типа и AQP-2». Если это так, то пожилые пациенты с несахарным диабетом должны реагировать на более низкие дозы десмопрессина, чем более молодые пациенты [12]. Из вышесказанного можно сделать вывод, что вопрос о причинах столь различной чувствительности пациентов к десмопрессину остается открытым.

Для подтверждения несахарного диабета пациенту проведены тесты с осмотической стимуляцией. Согласно их результатам, в ходе теста с водной депривацией (зафиксировано повышение натрия до 146,2 ммоль/л, осмоляльности крови до 300 мОсм/кг, при максимальной осмоляльности мочи 228 мОсм/кг) и инфузионной пробы с гипертоническим (3%) раствором (повышение натрия крови до 147,6 ммоль/л, повышение осмоляльности крови до 307 мОсм/кг, максимальная осмоляльность мочи — 337 мОсм/кг). На фоне функциональных проб при проявлениях обезвоживания со стороны внутренней среды в виде гипернатриемии и гиперосмоляльности отмечалась одновременная неспособность сконцентрировать мочу, о чем свидетельствовала ее низкая осмоляльность. Из перечисленного выше можно сделать вывод об отсутствии ремиссии несахарного диабета.

На пике обезвоживания (после окончания теста с водной депривацией) проведен тест с десмопрессином, синтетическим аналогом АДГ, активирующим V2R. После приема пациентом 0,1 мг десмопрессина per os измеренная осмоляльность мочи через 2 ч составила 431 мОсм/кг, через 4 ч — 634 мОсм/кг, что, согласно критериям дифференциальной диагностики, соответствует несахарному диабету центрального генеза.

Несмотря на значительное снижение потребности в препарате, симптомов передозировки десмопрессином не наблюдалось, отмена терапии в данном случае привела бы к нежелательным последствиям в виде появления полиурии-полидипсии, а также симптомов обезвоживания. Поэтому пациенту было рекомендовано продолжить терапию десмопрессином [13]. По причине индивидуальной чувствительности к препарату крайне важно определять продолжительность действия и потребность в препарате по выраженности симптомов жажды и полиурии у каждого пациента. В случае представляемого нами пациента наблюдаемая повышенная чувствительность к препарату может объясняться различными физиологическими и генетическими факторами, о которых мы упоминали ранее.

Основным условием успеха лечения десмопрессином является купирование симптомов заболевания: избыточной жажды и полиурии. Не следует рассматривать в качестве целей лечения достижение референсных интервалов лабораторных показателей, особенно относительной плотности мочи в каждой из проб анализа мочи по Зимницкому, поскольку не у всех пациентов с ЦНД на фоне отсутствия клинических проявлений заболевания достигаются нормальные показатели концентрационной функции почек (физиологическая вариабельность плотности мочи в течение дня, пожилой возраст, сопутствующая патология почек и др.) [1].

Учитывая, что подобранная минимально эффективная доза препарата обеспечивает оптимальное качество жизни, мы можем предположить, что у нашего пациента наблюдается высокая чувствительность к десмопрессину. При выписке пациенту рекомендован прием препарата по потребности, и обязательным условием являлось соблюдение питьевого режима по жажде.

Необходимо учитывать, что эндогенный АДГ активен 15 минут, при сохранении потребности в антидиурезе гормон должен быть секретирован дополнительно в нейрогипофизе. Десмопрессин же активен 8–12 ч, в течение которых он оказывает постоянное антидиуретическое действие, следовательно, выведение излишней жидкости затруднено, во избежание водной интоксикации следует ограничивать объем потребляемой жидкости [14].

Механизм развития водной интоксикации включает в себя задержку воды в почках и увеличение внеклеточной жидкости, что компенсируется повышенной экскрецией Na с мочой. Сочетание задержки воды и экскреции Na приводит к гипонатриемии [15].

С практической точки зрения нужно помнить о том, что при снижении потребности в десмопрессине вследствие высокой чувствительности пациента к терапии важно не допустить развития водной интоксикации, которая может быть опасна вплоть до летального исхода. При выписке пациента необходим строгий инструктаж: прием жидкости должен осуществляться только при жажде, количество выпитой за один прием жидкости не должно превышать 300 мл, также необходимо обсудить с пациентом возможность отсрочки последующей дозы препарата до возникновения симптомов жажды.

## ЗАКЛЮЧЕНИЕ

Таким образом, течение ЦНД в редких случаях может быть связано с изменением чувствительности к десмопрессину. Важным условием достижения компенсации и безопасного лечения несахарного диабета является подбор формы введения и дозы препарата, который позволит поддерживать максимально комфортное для пациента качество жизни.

## ДОПОЛНИТЕЛЬНАЯ ИНФОРМАЦИЯ

Источники финансирования. Работа выполнена по инициативе авторов без привлечения финансирования.

Конфликт интересов. Авторы декларируют отсутствие явных и потенциальных конфликтов интересов, связанных с содержанием настоящей статьи.

Участие авторов. Все авторы одобрили финальную версию статьи перед публикацией, выразили согласие нести ответственность за все аспекты работы, подразумевающую надлежащее изучение и решение вопросов, связанных с точностью или добросовестностью любой части работы.

Согласие пациента. Пациенты добровольно подписали информированное согласие на публикацию персональной медицинской информации в обезличенной форме в журнале «Проблемы эндокринологии».
